# Oxidative stress predicts cognitive decline with aging in healthy adults: an observational study

**DOI:** 10.1186/s12974-017-1026-z

**Published:** 2018-01-16

**Authors:** Ihab Hajjar, Salim S. Hayek, Felicia C. Goldstein, Greg Martin, Dean P. Jones, Arshed Quyyumi

**Affiliations:** Emory Univeristy, 1841 Clifton Road NE, Atlanta, GA 30329 United States

**Keywords:** Aging, Cognition, Oxidation, Inflammation, Glutathione, Cysteine

## Abstract

**Background:**

Redox signaling, which can be assessed by circulating aminothiols, reflects oxidative stress (OS) status and has been linked to clinical cardiovascular disease and its risk factors. These, in turn, are related to executive function decline. OS may precede the pro-inflammatory state seen in vascular disease. The objective of this study is to investigate the association between aminothiol markers of OS and inflammation in cognitive decline, especially in the executive cognitive domain which is highly susceptible to cardiovascular risk factors and is an important predictor of cognitive disability.

**Methods:**

The study design is that of a longitudinal cohort study within the setting of a large academic institution with participants being university employees (*n* = 511), mean age 49 years, 68% women, and 23% African-American. These participants were followed for four consecutive years with a yearly cognitive assessment conducted using computerized versions of 15 cognitive tests. Peripheral cystine, glutathione, their disulfide derivatives, and C-reactive protein (CRP) were measured.

**Results:**

Lower levels of glutathione at baseline was associated with a decline in the executive domain over 4 years (covariate-adjusted relative risk (RR) for glutathione = 1.70 (95% CI = 1.02–2.85), *p* = 0.04). Furthermore, a longitudinal decline in glutathione level was associated with a faster decline in the executive domain (*p* = 0.03). None of the other OS markers or CRP were linked to cognitive decline over 4 years.

**Conclusion:**

Increased OS reflected by decreased glutathione was associated with a decline in executive function in a healthy population. In contrast, inflammation was not linked to cognitive decline. OS may be an earlier biomarker that precedes the inflammatory phase of executive decline with aging.

## Background

Oxidative stress (OS) that is marked by the increased production of reactive oxygen species (ROS) has been implicated in both cardiovascular pathophysiology and in neurodegeneration [1–3]. OS has also been proposed as a fundamental driver of aging where increased production of ROS and/or decreased antioxidant defenses lead to macromolecular damage, impaired organ function, and development of disease [4]. However, clinical trials with non-specific antioxidants such as vitamins have failed to demonstrate benefit. This may be related to the fact that a focus on ROS is simplistic, as some ROS species such as superoxide anion radical, hydrogen peroxide, and nitric oxide also play an essential role in signaling and other physiological processes in the vascular system and the brain [5]. An alternative approach of OS as a disruption of redox signaling and control has been proposed [6].

Aminothiol-containing proteins are prone to oxidation-reduction reactions. Biological organization and several physiological functions are mediated by these switches in proteins [7]. In this organizational structure, glutathione functions as a major antioxidant in tissues, supporting the elimination of peroxides and detoxification of reactive aldehydes and other toxic chemicals. In the plasma, cysteine undergoes oxidation to its disulfide, cystine, which must be cleared from the circulation and reduced back to cysteine to prevent disruption of the thiol switching mechanisms [8]. Quantifying these circulating thiol proteins thus may provide a more accurate assessment of systemic OS allowing the investigation of its contribution to aging and chronic disease [9].

We have shown that increased OS as assessed by measuring circulating aminothiols is associated with cardiovascular risk factors, subclinical cardiovascular disease, and are strong predictors of incident adverse cardiovascular events and mortality [10–14]. Since cardiovascular risk factors are also associated with a decline in executive function, we hypothesized that aminothiol markers of OS will be predictive of cognitive decline in healthy adults without dementia [15, 16]. To test our hypothesis, we investigated the association between aminothiol markers of OS as well as the inflammatory marker, C-reactive protein (CRP), and cognitive decline, especially in the executive cognitive domain which is highly susceptible to cardiovascular risk factors in a healthy population free of significant cardiovascular disease or cognitive disorders.

## Methods

### Study description

This study was conducted in the Center for Health Discovery and Well Being at Emory University and Georgia Institute of Technology which recruited a cohort of institutional employees [17] (http://predictivehealth.emory.edu) as described previously [18]. The Emory University Institutional Review Board approved the protocols, and informed consent was obtained from all participants. Exclusion criteria were a history in the past year of hospitalization (except for accidents); severe psychosocial disorders; addition of new prescription medications to treat a chronic disease (except for changes in antihypertensive or antidiabetic agents); drug abuse or alcoholism; a current active malignant neoplasm; uncontrolled or poorly controlled autoimmune, cardiovascular, endocrine, gastrointestinal, hematologic, infectious, inflammatory, musculoskeletal, neurological, psychiatric, or respiratory disease; and any acute illness in the 2 weeks before the baseline studies. Physical measures (blood pressure, heart rate, dual-energy X-ray absorptiometry, body mass index, and treadmill testing), laboratory tests (metabolic, hematologic and inflammatory markers), cardiovascular function, health behaviors, medication profiles, mental health markers, and cognitive function were measured at yearly intervals.

### Cognitive assessment

Commonly employed versions of neuropsychological measures were administered via computer at baseline and then yearly for a total of four times, using a software developed by Aharonson and colleagues [19–21]. Cognitive tests included memory delayed recall, memory recognition, visual-spatial learning, spatial short-term memory, pattern recall, delayed pattern recall and recognition of pattern, executive function test, mental flexibility, digit symbol substitution test, forward and backward digit span, symbol spotting, and focused and sustained attention (computerized score: 0–100% correct adjusted for skill levels). The symbol spotting includes a set of 20 symbols, and the subject is asked to press the “+” symbol when the plus symbol appears. Other tests, such as executive function, included identifying the odd pattern by pressing the corresponding number of the pattern out of multiple patterns. The digit symbol substitution test included showing the subject a code for symbols and digits then the subject is instructed to substitute each symbol with the appropriate digit underneath it. Digit span instructs the subject to repeat a set of digits (forward or backward) after showing them the set of digits. A full description of this battery is available here [19–21]. Cognitive scores for cognitive domains were derived using principal component analysis with Varimax (orthogonal) rotation and Kaiser normalization to perform the exploratory factor analysis and was then followed with a confirmatory factor analysis by exploring the correlations and model fit of the derived factor-saved scores as reported previously in our reports [22, 23]. The factor analysis resulted in deriving three scores related to executive function, memory, and working memory. The distributions of the cognitive scores were extremely skewed to the right, as were the raw scores, suggesting a high-performance level of the participants (probably related to the high educational levels of the sample: 18.7 (standard error = 0.2 years) and a ceiling effect of the tests used. Test scores were divided in all the analyses at each visit into binomial variables: low vs high performance if a participant’s score was below the cut-off of the lowest quartile of the corresponding score at baseline. Factor analysis and cut-off were derived from the baseline sample of 601 participants.

### Markers of oxidative stress and inflammation

Plasma cysteine (CyS), its oxidized form cystine (CySS), glutathione, and its oxidized form glutathione disulfide (GSSG) were measured by high-performance liquid chromatography [6, 9]. Blood samples were drawn and transferred into pre-prepared Eppendorf tubes containing preservatives to retard auto-oxidation, centrifuged, and stored at − 80 °C for no more than 2 months. Sample collection and storage conditions have been previously described [24]. Analyses by high-performance liquid chromatography were performed after dansyl derivatization on a 3-aminopropyl column with fluorescence detection. Metabolites were identified by coelution with standards and quantified by integration relative to the internal standard, with validation relative to external standards. Higher levels of the oxidized derivatives and lower levels of the reduced forms represent increased OS. The coefficient of variation for cystine is 3.2% and glutathione 5%. Subjects also underwent venous blood collection for the measurement of the inflammatory marker, CRP via Multiplex kit (R&D Systems, Minneapolis, MN).

### Statistical analysis

We limited our analysis to those with both cognitive and OS and inflammatory cytokines assessments at baseline who had two separate cognitive assessments during follow-up (*n* = 511). To estimate the age, gender, and race-adjusted change in the calculated cognitive scores, we performed multiple regression analysis after adjusting for these three demographic variables. We provide the rate as a unit of change in the calculated score per year (unit/year). Baseline correlations between OS markers and cognitive scores were performed using regression analyses. Cognitive scores and aminothiol levels were included as discrete variables due to their skewness in the longitudinal analysis. We used binomial regression with generalized estimating equations (GEE) for repeated discrete measures. GEE is appropriate for binary repeated correlated measures, and it allows for the estimation of risk in the population [25, 26]. The cumulative relative risk of having cognitive decline (below the cut-off derived from the baseline score) was calculated during the follow-up [27]. To assess the relation between changes in OS and cognition, we first calculated a within-subject slope for each measure (yearly change) and then performed multiple standardized regressions between the rate of change in OS markers and cognitive measures. All analysis models were adjusted for age, gender, race, education, systolic blood pressure, statin, and antihypertensive therapy. All analyses were conducted in SAS (V9.4, Carey, NC).

## Results

Our final analytical sample was 511 (85% of the 601 who had cognitive assessment at baseline). The mean age at baseline was 48 years, 68% were women and 24% were African-American, Table 1. The median number of follow-up visits with OS and cognitive assessments was 4, and the mean follow-up time was 49.4 months. Pearson correlation coefficients between CRP and OS markers were − 0.07 with glutathione (*p* = 0.07), − 0.008 with GSSG (*p* = 0.84), 0.06 with CYS (*p* = 0.18), and 0.18 with CYSS (*p* < 0.001). Over the study period, the age-, gender-, race-adjusted decline in the executive domain was 0.25 unit/year (95% CI 0.21–0.30), in the memory domain was 0.23 unit/ year (95% CI 0.19–0.27), and in the working memory domain was 0.23 unit/year (95% CI 0.19–0.28).

**Table 1:** Baseline characteristics (*n* = 511) of the participants with OS markers and at least two cognitive evaluations

	Mean or count	Standard error or %
Age, years	49.1	0.5
Women	348	68%
Race: White/African-American	366/117	72%/23%
Body mass index, kg/m^2^	27.68	0.27
Years of education	18.91	0.2
Systolic blood pressure, mmHg	121	1
Diastolic blood pressure, mmHg	76	0.5
Hypertension	156	31%
Hypercholesterolemia	96	19%
Diabetes mellitus, type 2	20	4%
CRP, mg/L	0.2	0.02
Markers of oxidative stress		
GSH, μmol/L	4.4	0.1
GSSG, μmol/L	0.06	0.01
CYS, μmol/L	9.3	0.09
Cystine, μmol/L	85.3	0.8
Cognitive function score		
Memory domain	65.7	0.7
Executive function domain	83.6	0.2
Working memory domain	55.0	0.4

*Cys* cysteine, *CYSS* cystine, *GSH* glutathione, *GSSG* glutathione disulfide

Baseline association between OS and cognitive performance: At baseline, higher GSSG levels, indicative of higher OS, were associated with worse performance on the memory (*p* = 0.009) and working memory (*p* < 0.0001) domains but with better performance on the executive function domain (*p* < 0.0001). None of the other markers were associated with baseline memory domains (Table 2). CRP tended to be associated with baseline working memory (*p* = 0.06) but not memory or executive function.

**Table 2:** Baseline cross-sectional associations between cognitive scores and OS markers/CRP

Cognitive domain	Marker	Unadjusted		Multivariate	*p* value
Executive domain				*β*	
	CRP	0.24	0.63	0.109	0.86
	CyS	− 0.088	0.27	− 0.11	0.24
	CySS	− 0.013	0.16	− 0.0028	0.81
	GSSG	16.35	< .0001	17.91	< .0001
	GSH	0.18	0.12	0.23	0.095
Memory domain					
	CRP	0.14	0.9	0.19	0.88
	CyS	0.13	0.46	0.29	0.14
	CySS	− 0.0047	0.82	0.013	0.63
	GSSG	− 19.17	0.009	− 22.25	0.0035
	GSH	0.020	0.94	− 0.048	0.877
Working memory domain					
	CRP	− 2.27	0.0028	− 1.67	0.063
	CyS	− 0.20	0.089	− 0.19	0.14
	CySS	− 0.0079	0.57	0.0091	0.60
	GSSG	− 30.78	< .0001	− 33.67	< .0001
	GSH	− 0.22	0.22	− 0.47	0.020

All models were adjusted for race**,** age**,** gender**,** systolic blood pressure, body mass index, education, statin, and antihypertension medication

*β* is derived from the regression analyses of the oxidative stress marker vs cognitive function

*CRP* C-reactive protein, *Cys* cysteine, *CYSS* cystine, *GSH* glutathione, *GSSG* glutathione disulfide

**Fig. 1:**
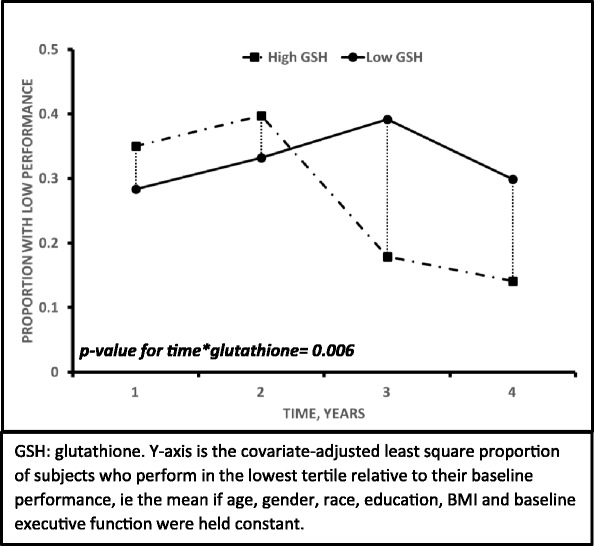
Association of baseline glutathione with the proportion of subjects with decreased executive function over the 4-year period

Relationship between baseline OS and longitudinal cognitive decline: Lower levels of glutathione at baseline, reflecting increased OS, were associated with a greater likelihood of having a lower score on the executive domain over 4 years [covariate-adjusted relative risk (RR) = 1.70 (95% CI = 1.02–2.85), *p* = 0.04]. In those with high (> 1 SD units) glutathione levels at baseline, the number of participants performing below the cut-off (bottom quartile score at baseline) for the executive domain decreased from 35% at baseline to 14% at year 4. In contrast, that number increased from 28 to 30% in those with low glutathione levels at baseline. The difference between the baseline high vs low glutathione levels was statistically significant (*p* value for time*baseline glutathione = 0.006) as shown in Fig. 1. Baseline CRP, CYS, CYSS, and GSSG levels were not associated with the change in executive function or other memory domains.

Change in markers of oxidative stress and cognitive decline: Longitudinal decreases in glutathione level (*β* = − 1.7, *p* = 0.03) were associated with a greater decline in the executive domain, after adjusting for age, gender, race, education, blood pressure, use of statin, and antihypertensive therapy (Table 3). For every unit decline in glutathione, executive function declined by 1.7 units (*p* = 0.03), and this was not true for any other OS marker.

**Table 3:** Unadjusted and adjusted population risk for having cognitive decline to below the baseline cut-off for the corresponding cognitive domain during the study visits [1–4]

	Univariate			Multivariate*				
	RR	95% CI		*p* value	RR	95% CI		*p* value
Cys								
Memory domain	0.54	0.20	1.46	0.22	0.61	0.20	1.87	0.39
Executive function domain	1.11	0.66	1.86	0.70	1.08	0.65	1.78	0.78
Working memory domain	0.68	0.42	1.08	0.10	0.56	0.35	0.90	0.05
CYSS								
Memory domain	0.89	0.50	1.58	0.69	0.92	0.51	1.66	0.78
Executive function domain	0.96	0.60	1.52	0.86	0.82	0.51	1.31	0.41
Working memory domain	1.15	0.72	1.85	0.56	1.07	0.65	1.76	0.78
GSH								
Memory domain	0.83	0.43	1.58	0.57	0.64	0.31	1.32	0.23
Executive function domain	1.82	1.10	3.01	0.02	1.70	1.02	2.85	0.04
Working memory domain	0.97	0.60	1.57	0.91	1.08	0.63	1.86	0.78
GSSG								
Memory domain	1.15	0.72	1.85	0.56	1.07	0.65	1.76	0.78
Executive function domain	1.18	0.65	2.15	0.59	1.56	0.81	3.04	0.19
Working memory domain	0.83	0.43	1.58	0.57	0.64	0.31	1.32	0.23
CRP								
Memory domain	1.43	0.61	3.34	0.41	0.94	0.59	1.50	0.94
Executive function domain	1.13	0.63	2.05	0.68	0.84	0.45	1.54	0.84
Working memory domain	1.05	0.67	1.64	0.83	0.94	0.59	1.50	0.94

*Models adjusted for race, age, gender, systolic blood pressure, body mass index, education, statin, and antihypertension medication use and baseline performance

*CRP* C-reactive protein, *CYS* cysteine, *CYSS* cystine, *GSH* glutathione, *GSSG* glutathione disulfide

## Discussion

This study demonstrates that decreased circulating levels of glutathione predict the age-related decline in the executive domain, an area of cognition that is particularly susceptible to cardiovascular disease. In addition, higher levels of the oxidized glutathione, GSSH, are related to lower performance on both memory and working memory but better executive function. Most uniquely in our analysis, we demonstrate that age (or time)-related changes in OS correlate with the age-related decline in the executive domain. Extending our previous reports relating aminothiol levels with subclinical vascular disease and incident cardiovascular events, we now demonstrate that circulating aminothiol levels serve as a biomarker of change in executive function [11].

The existence of a role for OS in vascular disease and cognitive aging has been suggested, but most studies have so far focused on ROS [28]. Our findings indicate that glutathione may predict aging-related decline in executive function. This is important because decline in executive function is a better predictor, more so than memory, of future functional loss in normal controls and those with mild cognitive impairment or dementia.

Use of free radical-scavenging antioxidants such as vitamin E has failed to show a consistent benefit on cognitive preservation and has been linked to increased cardiovascular mortality [29, 30]. However, vitamin E supplementation can *lower* glutathione levels through its effect on the glutathione S-transferase and may potentially exacerbate OS [31]. Our results demonstrating the link between aminothiol markers and cognitive preservation may offer an explanation for the inconsistent findings with vitamin E supplementation trials on cognitive outcomes.

Increased protein oxidation has been observed in the brains of persons with Alzheimer’s disease, especially in regions that are rich in amyloid peptide [32]. The underlying mechanisms remain unknown, although increased OS is thought to be an initial trigger [33]. Glutathione plays a key role in the antioxidant defense of neuronal cells, and circulating glutathione levels are reduced in AD [34]. Our findings show that the plasma glutathione levels predict future decline in cognition. Moreover, individuals who experience decreases in glutathione level over time also have a greater decline in their cognition. These findings potentially offer new targets for the prevention and treatment of cognitive loss with aging, especially that related to executive function where no therapy is currently available.

In the brain, glutathione synthesis varies by cell type and depends on its ability to use available extracellular glutathione precursors. Neurons rely on the presence of extracellular cysteine as a precursor for glutathione [35]. Glutathione is also released from astrocytes [36–40] and can be imported from blood into the brain through the blood-brain barrier [41, 42] In particular, a sodium-dependent glutathione transporter has been identified in the brain capillaries [43] and brain endothelial cells [44]. In disease states, a reduction in brain glutathione occurs with aging, Parkinson’s disease [45, 46], and Alzheimer’s disease [47]. This provides a biological explanation for our observations of the association between glutathione and cognition.

We did not demonstrate an association between CRP and cognitive decline in this population, apart from the baseline association with working memory performance where the association was borderline significant. Increased CRP, a marker of increased systemic inflammation, may occur later in the evolution of cognitive decline with aging and in neurodegeneration [48]. Further, peripheral CRP is a surrogate marker but not causative inflammatory mediator, thus we cannot exclude the possibility that decreased serum levels of glutathione are mirrored by simultaneous increased levels of inflammatory mediators in the CNS which in turn are linked to cognitive decline.

Strengths of our study are the inclusion of healthy disease-free middle-aged individuals, the assessment of blood aminothiols as unique markers of OS, and detailed cognitive testing. The limitation of our study is relatively short follow-up period and the lack of brain imaging. In addition, peripheral GSH/GSSG as a biomarker for oxidative stress is not as good as tissue measures and hence may be missing significant other associations with cognition. The differential effects between peripheral and tissue may explain the lack of coupling between GSH and GSSG. Finally, additional oxidative stress markers from lipid and nucleic acid were not measured.

## Conclusion

OS reflected by a low or a progressive decrease in glutathione levels is associated with a decline in executive function with aging. The role of OS in cognitive decline offers further insights into the processes of cognitive aging and the link with vascular risk factors and warrants further investigation.
